# Quantifying the Human Health Benefits of Using Satellite Information to Detect Cyanobacterial Harmful Algal Blooms and Manage Recreational Advisories in U.S. Lakes

**DOI:** 10.1029/2020GH000254

**Published:** 2020-08-25

**Authors:** Signe Stroming, Molly Robertson, Bethany Mabee, Yusuke Kuwayama, Blake Schaeffer

**Affiliations:** ^1^ School of Foreign Service Georgetown University Washington DC USA; ^2^ Resources for the Future Washington DC USA; ^3^ Office of Research and Development United States Environmental Protection Agency Research Triangle Park NC USA

**Keywords:** Value of information, Water quality, Harmful algal blooms, Recreational advisories, Human health

## Abstract

Significant recent advances in satellite remote sensing allow environmental managers to detect and monitor cyanobacterial harmful algal blooms (cyanoHAB), and these capabilities are being used more frequently in water quality management. A quantitative estimate of the socioeconomic benefits generated from these new capabilities, known as an impact assessment, was missing from the growing literature on cyanoHABs and remote sensing. In this paper, we present an impact assessment framework to characterize the socioeconomic benefits of satellite remote sensing for detecting cyanoHABs and managing recreational advisories at freshwater lakes. We then apply this framework to estimate the socioeconomic benefits of satellite data that were used to manage a 2017 cyanoHAB event in Utah Lake. CyanoHAB events on Utah Lake can pose health risks to people who interact with the blooms through recreation. We find that the availability of satellite data yielded socioeconomic benefits by improving human health outcomes valued at approximately $370,000, though a sensitivity analysis reveals that this central estimate can vary significantly ($55,000–$1,057,000 in benefits) as a result of different assumptions regarding the time delay in posting a recreational advisory, the number of people exposed to the cyanoHAB, the number of people who experience gastrointestinal symptoms, and the cost per case of illness.

## Key Points

- Cyanobacterial harmful algal blooms (cyanoHABs) are a human health risk that is typically mitigated by recreational advisories and warnings
- We present a framework to quantify the socioeconomic benefits of using remote sensing technology to test for the presence of cyanoHABs
- Satellite technology yielded between $55,000 and $1,057,000 in socioeconomic benefits associated with improved human health outcomes

## 1 Introduction

Cyanobacterial harmful algal blooms (cyanoHAB) have received increasing attention from researchers and policymakers due to potential negative impacts on human and ecological health. A cyanoHAB is an environmental event that occurs when cyanobacterial algal populations achieve sufficiently high density resulting in negative environmental or health consequences (Smayda, 1997). Recently, there have been significant advances in satellite remote sensing that environmental managers use to detect and manage cyanoHABs (Hunter et al., 2017). These capabilities are being used more frequently to manage recreational water bodies, reservoirs (Schaeffer et al., 2018), commercial and recreational fishing, and aquaculture (Gernez et al., 2017; Snyder et al., 2017). While there is anecdotal evidence that the availability of satellite data on cyanoHABs has led to improved management decisions, there are no existing quantitative estimates of the improved outcomes generated from these changes. We define “improved outcomes” as those that provide socioeconomically meaningful benefits, that is, measurable increases in well‐being or stability for people in a community, such as reductions in human health impacts and health care expenses. Generating estimates for socioeconomic benefits created by cyanoHAB detection and monitoring programs can help demonstrate the value of their work in socioeconomically meaningful terms and help remote sensing experts target improvements in these data products in ways that maximize their benefits to society.

In this paper, we leverage an impact assessment framework to characterize the socioeconomic benefits of satellite remote sensing for detecting cyanoHABs and managing recreational advisories at freshwater lakes. We first collect and summarize information on how satellite data are currently used by decision makers to take actions that seek to reduce the negative societal impacts of cyanoHABs. We obtain most of this information from guidance documents published by state and federal government agencies and from interviews with researchers who provide satellite data products to decision makers as well as with the decision makers themselves. Using this background information on decision contexts, we leverage an impact assessment framework to quantify the socioeconomic benefits of satellite remote sensing for detecting cyanoHABs and managing recreational advisories at freshwater lakes. This framework requires an impact assessment that documents (a) differences between the actions that decision makers take when the satellite information is available and the actions they take when satellite information is unavailable and (b) whether socioeconomically meaningful outcomes, such as those relating to human or ecological health, are improved when decision makers have access to satellite information.

We then apply this impact assessment framework to estimate the socioeconomic benefits of satellite data that were used to manage a 2017 cyanoHAB event in Utah Lake. In this application, we find that the availability satellite data yielded socioeconomic benefits in the form of improved human health outcomes valued at $370,000, though a sensitivity analysis reveals that this central estimate can vary significantly as a result of different assumptions regarding the time delay in posting a recreational advisory, the number of people exposed to the cyanoHAB, the number of people who present with gastrointestinal symptoms, and the cost per case of illness.

These results indicate that improving decision contexts with satellite data can provide some socioeconomic benefit to cities and states by improving human health outcomes following a cyanoHAB event. Quantifying the socioeconomic benefits of satellite data in applications like detecting and monitoring cyanoHABs is important because it demonstrates return on investment in satellites and associated data production activities, helps shape priorities across potential future investments in satellite data applications, and informs decision makers who are currently not using the data but are considering using it in future decisions. Quantification of these benefits also helps Earth scientists express the value of their work in socioeconomically meaningful terms.

## 2 Background on CyanoHABs

CyanoHABs occur in both brackish and freshwater systems when colonies of cyanobacteria grow rapidly and pose a threat to their host ecosystems. Most freshwater blooms occur where water is warm, still, and nutrient rich (Paerl & Otten, 2013). Freshwater cyanoHABs are most often observed in late summer or early fall, in low‐wind conditions, in shallow water bodies that collect agricultural runoff or have high levels of nutrient pollution. Certain bodies of water are at higher risk for cyanoHABs due to depth, temperature, and exposure to sunlight. These bodies of water might experience algal blooms with higher frequency. Once a bloom occurs, it may persist for months, even after nutrient resources within the water body have been depleted (Paerl & Otten, 2013). These cyanoHABs can have negative impacts on the health of humans and animals (Backer et al., 2013, 2015). CyanoHABs can produce a variety of harmful cyanotoxins, including 246 variants of microcystin, seven variants of anatoxin, cylindrospermopsin, and saxitoxins (Loftin et al., 2016; Meriluoto et al., 2017; U.S. EPA, 2014). Microcystin, the most frequently occurring and widespread cyanotoxin, is a liver toxin (World Health Organization, 2003). Anatoxin‐a and saxitoxins affect the nervous system, while cylindrospermopsin may cause kidney or liver failure (World Health Organization, 2003). The toxicity of cyanobacterial blooms is highly variable and is unrelated to the specific type of cyanobacteria present. In a global study, about 60% of cyanobacteria samples contained toxins, with toxicity of those samples ranging from 25% to 95% (Nabout et al., 2013; World Health Organization, 2003).

Toxic and nontoxic cyanoHABs have been documented in freshwater systems on all seven continents (Clark et al., 2017). The *National Lakes Assessment*, a statistical survey of the condition of lakes, ponds, and reservoirs in the United States, detected microcystin in 39% of lakes in the United States in 2012—a 9.5% increase from 2007—although microcystin was rarely at moderate to high levels of exposure risk (USEPA, 2016). Toxic and nontoxic cyanoHABs alike have the potential to increase in frequency (Clark et al., 2017), extent (Urquhart et al., 2017), or magnitude (Mishra et al., 2019) due to climate change because warmer temperatures, salinization of water sources, and more frequent extreme rainfall events tend to benefit cyanobacterial bloom production (Paerl & Otten, 2013).

### 2.1 The Socioeconomic Impacts of CyanoHABs

One of the primary mechanisms through which cyanoHABs cause human health impacts is by contaminating drinking water. Public water systems are generally capable of preventing intact cyanobacterial cells or lower levels of cyanotoxins from entering drinking water, but during severe bloom events, when there are high levels of cyanobacterial cells or cyanotoxins near drinking water intakes (Falconer & Humpage, 2005; Hoeger et al., 2005; Kouzminov et al., 2007; Qin et al., 2010; Ueno et al., 1996), public water systems may struggle to provide quality drinking water to the public (USEPA, 2019). There have been two recent events in Ohio that reflect the risk of cyanotoxins contaminating drinking water. In 2013, the Ohio Environmental Protection Agency (Ohio EPA) placed Carrol Township, a community of 2,000 residents, under a “Do Not Drink” advisory after testing revealed dangerous levels of toxins in the local drinking water facility (Jochem, 2017). In Toledo, high levels of microcystin in the water supply in 2014 prompted city officials to issue a drinking water advisory for the entire city, causing 500,000 residents to rely on bottled water for 3 days (Fitzsimmons, 2014). The Centers for Disease Control and Prevention (CDC) reports 110 cases of illness attributable to cyanotoxins from the Toledo event, with acute gastrointestinal illness as the most common symptom (Benedict et al., 2017).

Another important exposure route to toxic cyanobacterial blooms is through food consumption. Toxins may bioaccumulate in seafood from both marine and freshwater environments (Ibelings & Chorus, 2007; Poste et al., 2011; Schmidt et al., 2013). People and animals may be exposed to cyanotoxins by eating fish or shellfish from water bodies experiencing a cyanobacterial bloom. Some studies indicate that cyanotoxins also have the potential to be absorbed by agricultural crops irrigated with cyanoHAB‐affected water, but research on associated health risks is extremely limited (Corbel et al., 2014; Seungjun et al., 2017).

Toxic cyanobacterial blooms can also pose human health risks through recreation in cyanoHAB‐affected water. Recreational users may be exposed to cyanobacterial blooms through accidental ingestion of cyanoHAB‐affected water while swimming, inhalation of water vapor while boating or water skiing, and direct dermal contact while swimming or wading. Anecdotal evidence of allergic reactions due to dermal contact with cyanobacteria in recreational waters is frequently reported, but few scientific studies exist (World Health Organization, 2003). In one lake in Michigan, Jones (2019) found that a cyanoHAB was responsible for decreased birth weight in infants born in Kalamazoo and Barry counties; these cases of low birth weight led to approximately $768,500 in annual hospitalization costs in the two counties. Jones (2019) did not definitively conclude that recreation was the only pathway for contact with the cyanoHAB but suggested that it was likely a key contributor. The popularity of freshwater recreation in the United States means large numbers of people are potentially exposed to cyanobacteria or cyanotoxins each year.

Even nontoxic blooms may generate negative socioeconomic impacts, causing hypoxia and increasing water turbidity, which degrades ecosystem services by depriving aquatic plants of sunlight for photosynthesis and aquatic organisms of oxygen for respiration. CyanoHABs may also generally degrade the aesthetic quality of a water body, which negatively affects public perception of a water body's suitability for recreation (World Health Organization, 2003; Zhang & Sohngen, 2018). Chronic hypoxia and turbidity can also negatively impact recreational fishing industries and waterfront property values (Wolf et al., 2017; Wolf & Klaiber, 2017; Zhang & Sohngen, 2018). Increased turbidity and total organic carbon may also increase costs of producing potable water, increase disinfection byproduct production, and create taste and odor issues for drinking water (Cheung et al., 2013).

### 2.2 Satellite Remote Sensing of CyanoHABs

Detecting and monitoring cyanoHABs in recreational freshwater is a complex task. Many states practice reactive cyanoHAB management strategies, sending teams out after events are reported by recreational users or park managers. More proactive environmental quality departments use weekly or monthly in situ visual observations for cyanoHAB detection and monitoring. In some lakes, in‐water sensors have been installed on buoys to provide real‐time water quality monitoring.

**Figure 1:**
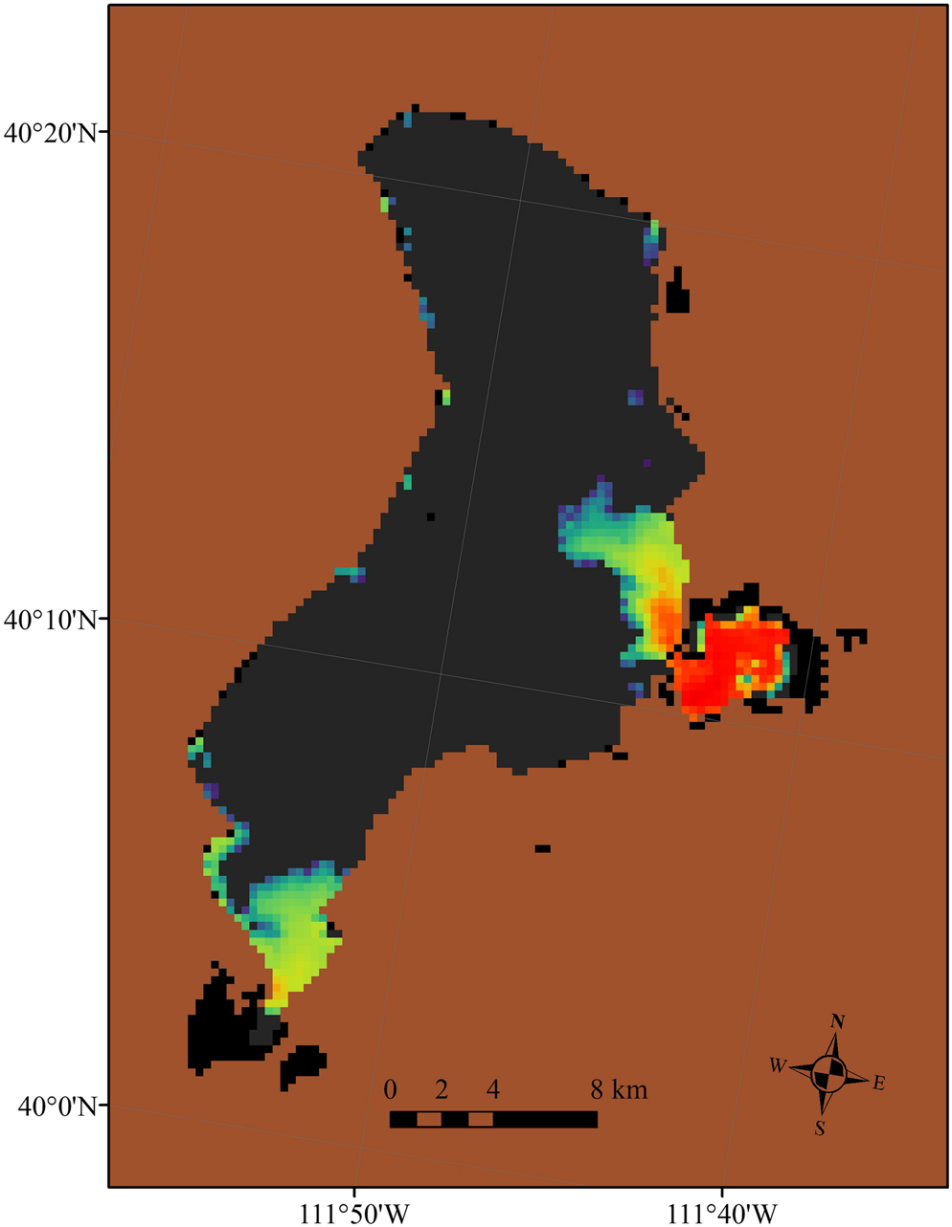
Copernicus Sentinel‐3 daily satellite image on 21 June 2017 of Utah Lake, Utah from the CyAN project*.* Note: Brown pixels are land, gray pixels in the lake indicate no detection of cyanobacteria, black pixels are quality controlled for clouds, straylight, or glint. Cool blue and purple colors are low concentrations of cyanobacterial biomass and warm colors of yellow, orange, and red are elevated concentrations of cyanobacteria.

Some states have incorporated satellite remote sensing information into their methods for monitoring local water. Briefly, water quality parameters, such as cyanobacteria concentration, are derived by satellite through direct solar radiation entering the water column and absorbing or scattering light, depending on the types and concentrations of constituents within the water column (Schaeffer et al., 2013). Most cyanobacteria contain phycocyanin used to detect the presence of cyanobacteria spectrally with an absorption feature near 620–630 nm and a peak near 650 nm (Kutser, 2009). A more exhaustive review of satellite uses for water quality can be found in the International Ocean‐Colour Coordinating Group Report 17 (IOCCG, 2018). Various satellite data sources have a range of spatial, temporal, and spectral resolutions, which balance trade‐offs with other geophysical considerations such as ground resolution and revisit time (Mouw et al., 2015). Satellites have many limitations to consider such as interference from cloud cover and detection for only the upper depths of the water column (Coffer et al., 2020). Only water bodies of sufficient size and shape may be considered in order to accommodate the spatial resolution of the satellite data, where the ability of a satellite to resolve a water body depends on the ideal pixel size of the sensor and on the combined size and geometry of the water body (Clark et al., 2017). An example of cyanobacterial biomass derived from satellite imagery is provided in Figure 1.

The first reported application of satellite remote sensing for the detection of harmful algal blooms was in 1972 using United States Geological Survey's (USGS) Landsat 1 (Murphy et al., 1975), and the first report, known to the authors, of satellite remote sensing detecting cyanobacteria was in 1980 from the USGS Landsat 2 in the Baltic Sea (Ulbricht, 1983). In 2015, the US Environmental Protection Agency (USEPA), National Aeronautics and Space Administration (NASA), USGS, and National Oceanic and Atmospheric Administration (NOAA) collaboratively launched the Cyanobacterial Assessment Network (CyAN) a multiagency research project with the long‐term goal of using historical and current satellite data to develop an early warning indicator system to detect cyanoHABs in large freshwater lakes and reservoirs across the United States (Schaeffer et al., 2015). CyAN uses the Cyanobacteria Index algorithm as described in Lunetta et al. (2015), converted to an estimate of cyanobacterial cells per milliliter of water, derived from the European Space Agency Medium Resolution Imaging Spectrometer (MERIS) on the Envisat satellite and transferable to the Sentinel‐3 Ocean Land Colour Instrument (OLCI) from 2016 onward. CyAN satellite data are distributed to federal, state, and local partners through a publicly available mobile application (Schaeffer et al., 2018) and directly to state agencies at the time of writing. It is important to note that CyAN cannot directly detect toxins or if the concentration of those toxins poses a human health risk (Stumpf et al., 2016).

## 3 Decision Contexts for CyanoHAB Detection and Management

According to users in the cyanoHAB management community, satellite information allows for more nuanced decision making regarding cyanoHABs and can even provide forecasts that give decision makers advanced warning of potential blooms. Satellite information can show environmental managers where blooms begin to form and the trajectories and patterns in which they spread through a given water body. This can help the managers better understand each water body as a system and perhaps anticipate bloom behavior in the future. Satellite imagery may also serve as a valuable historical record of cyanoHAB events in a given lake or as an initial “screen,” triggering in situ testing of water sources that would otherwise go unmonitored (Schaeffer et al., 2019).

The United States has no binding federal regulations that define acceptable levels of cyanobacteria in recreational freshwater lakes or rivers or establish a uniform strategy for cyanoHAB monitoring. USEPA has released ambient water advisories for microcystin and cylindrospermopsin and provides recommendations for cyanobacteria and cyanotoxin monitoring in recreational waters (USEPA, 2019), but ultimately, individual states are responsible for developing their own cyanoHAB monitoring and response strategies (USEPA, 2019). State and local environmental managers and public health officials rely on the health thresholds provided by the World Health Organization (WHO), the draft guidelines provided by USEPA or their own established thresholds for cyanobacterial density or cyanotoxin presence to determine whether to issue recreational advisories to the public. Increasingly, states are turning to remotely sensed information from satellites to supplement their cyanoHAB monitoring methods and inform advisory decisions. Some state and local management agencies have issued guidance documents that describe how remote sensing is incorporated into their cyanoHAB detection strategy and how the information influences policy decisions.

**Figure 2:**
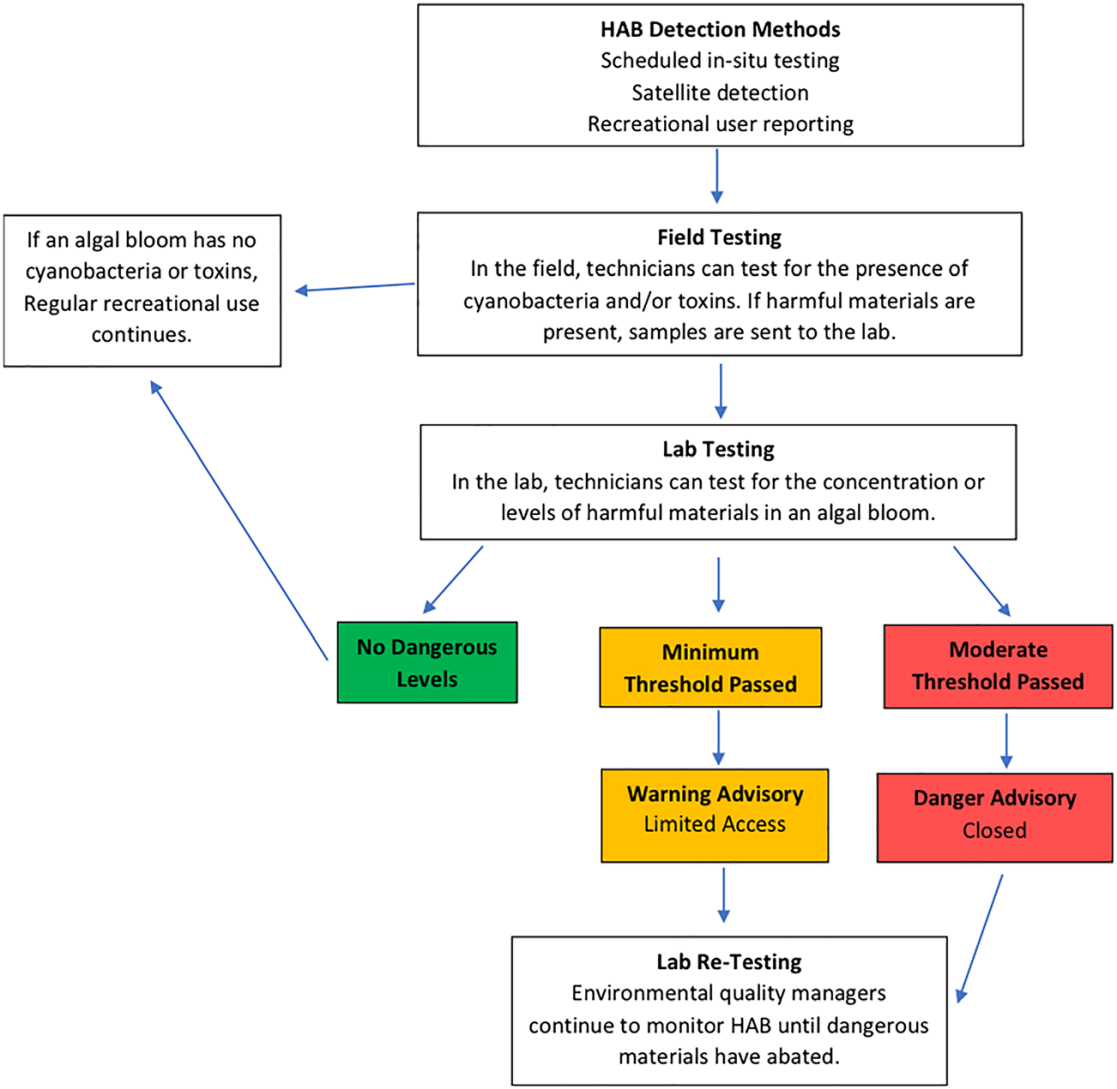
A representative decision process for detecting and responding to cyanobacterial cyanoHABs in freshwater recreational lakes.

Environmental and park managers typically have several different options when it comes to addressing a cyanoHAB in a recreational area. They can inform people that there is an increased risk of cyanoHABs during a given season, they can post warnings in specific areas, or they can close off access to ponds and lakes that are experiencing a cyanoHAB. Increased specificity regarding the timing and extent of cyanoHAB risk can make the choice between these options clearer. Figure 2 illustrates a typical process by which a state agency makes decisions about managing freshwater recreational areas using information on cyanoHABs. This figure is based on real decision maps provided by state regulatory agencies and is streamlined to demonstrate the key components of the cyanoHAB advisory decision‐making process that are shared among states. Specific thresholds vary between states, but environmental managers generally have two or three levels of action available based on the level of toxic materials revealed during lab tests.

Ohio, California, Florida, and Utah provide excellent examples for illustrating the variety of decision‐making contexts that rely on remotely sensed information on cyanoHABs. We summarize each of these contexts in turn below.

### 3.1 Ohio

Ohio has a well‐developed strategy for detecting and responding to cyanoHABs because of the prevalence of cyanoHABs in Lake Erie. With an average depth of only 62 feet, Lake Erie is the shallowest of the Great Lakes. Lake Erie's western basin is even shallower, with an average depth on only 24.1 feet, rendering it particularly susceptible to temperature fluctuations and a source of some of the most intense cyanoHABs in the lake (Bingham et al., 2015). In addition to regular sampling and reports from recreational users, Ohio has formalized the use of satellite imagery to assist in cyanoHAB detection. In 2009, NOAA's National Centers for Coastal Ocean Science (NCCOS) launched its cyanoHAB Forecasting Bulletin, a satellite imagery‐based weekly forecast for cyanoHAB events in Lake Erie, as well as seasonal projections (NOAA‐GLERL, n.d.). This report has proven critical in promptly identifying cyanoHABs in the state. As of 2018, NOAA reported the bulletin has close to 3,000 subscribers, including Ohio EPA staff, water supply managers, researchers, parks management, environmental groups, news media, and local citizens.

Ohio's unified statewide approach to addressing cyanoHABs in recreational waters is described in a 2016 document titled *Harmful Algal Bloom Response Strategy for Recreational Waters*, issued by the Ohio Department of Health, Ohio Department of Natural Resources, and Ohio EPA (State of Ohio, 2016). When a cyanoHAB is detected or suspected in Ohio, tests are conducted to determine toxicity levels. These tests typically take 1 and 2 days. Different concentrations of harmful toxins result in different advisory actions. General signage is used at any recreational body of water that has previously experienced a cyanoHAB. The signage describes what a bloom looks like and the symptoms it can cause. When the lowest advisory thresholds have been surpassed, warning signs are placed around the contaminated water that advise against children, pregnant women, and pets touching the water. The highest levels of contaminations prompt an elevated recreational public health advisory, which warns recreational visitors against making any contact with the water.

### 3.2 California

For California, satellite imagery was a crucial tool to facilitate cyanoHAB monitoring even before the CyAN network was developed (SFEI & ASC, n.d.). California's Surface Water Ambient Monitoring Program (SWAMP) partnered with NOAA to routinely monitor larger lakes in the state via satellite remote sensing. The San Francisco Estuary Institute (SFEI) has created an online portal through which environmental managers or the general public may access satellite‐derived water quality information for lakes across the entire state (SFEI & ASC, n.d.). The state's monitoring strategy now is supported by CyAN satellite monitoring for cyanoHABs and a central website where individuals can report events to the state.

When Californian environmental managers detect a cyanoHAB, they refer to the Office of Environmental Health and Hazard Assessment (OEHHA) action thresholds for cyanotoxins determined by the state. The thresholds specify maximum levels of cyanotoxins permissible for different water uses such as human recreation, fish consumption, and acute water intake for pets and livestock (Anderson‐Abbs et al., 2016). The state also provides guidance documents to local regulators on how to respond to cyanoHAB events and protect the public. The documents provide specific thresholds that inform environmental managers on when to post recreational advisories and how to educate the public more generally on the dangers of toxic blooms. In California, there are three levels of advisory action that are prompted by escalating toxin thresholds: caution, warning, and danger (State of California, 2018).

### 3.3 Utah

Utah's Department of Environmental Quality (Utah DEQ) uses satellite data to monitor for cyanoHABs in lakes across the state, especially as an initial screening system to trigger in situ testing of water bodies generally too remote to sample regularly (Ben Holcomb, Utah Department of Environmental Quality, personal communication, 27 July 2018). When there is a visible or suspected bloom, Utah DEQ checks for the presence of cyanobacteria or cyanotoxins via field tests. If those tests are positive, samples are sent to the lab for more sensitive tests of cyanotoxin concentrations or cyanobacterial cell densities. Like other states, Utah has a tiered response system that maps to different toxicity levels in a cyanoHAB. A moderate concentration of harmful substances prompts a “warning advisory,” while a higher concentration prompts a “danger advisory” (Butler et al., 2016).

Environmental managers first began using satellite data from the CyAN project to detect and monitor cyanoHABs in June 2017 (Schaeffer et al., 2015). Less than a month later, satellite data detected a bloom developing in Provo Bay in Utah Lake, prompting water quality managers to send out new teams to confirm the presence of a cyanobacterial bloom. More recently, satellite images were used in 2018 to detect cyanoHAB events in Panguitch Lake and Utah Lake that prompted warning advisories to be posted. Satellite images have also been used to provide greater detail on cyanoHAB development after cyanoHABs were detected by in situ testing. The application of satellite data to the cyanoHAB event in Utah Lake during the summer of 2017 is the case used in the application in section 5 of this paper to estimate the value of remote cyanoHAB monitoring.

## 4 Impact Assessment Framework

To quantify the socioeconomic benefits of satellite information when it is used in decisions, such as those involved in managing cyanoHABs in freshwater recreational lakes, we identify differences between the actions that decision makers take when the satellite information is available and the actions they take when satellite information is unavailable. In addition, we determine whether socioeconomically meaningful outcomes, such as those relating to human or ecological health, are improved when decision makers have access to satellite information. In other words, we compare outcomes that are realized in the current state of the world, in which satellite data on cyanoHABs are available (i.e., our “reference case”) to outcomes that are realized in an alternate state of the world in which the satellite data are not available (i.e., our “counterfactual case”).

**Figure 3:**
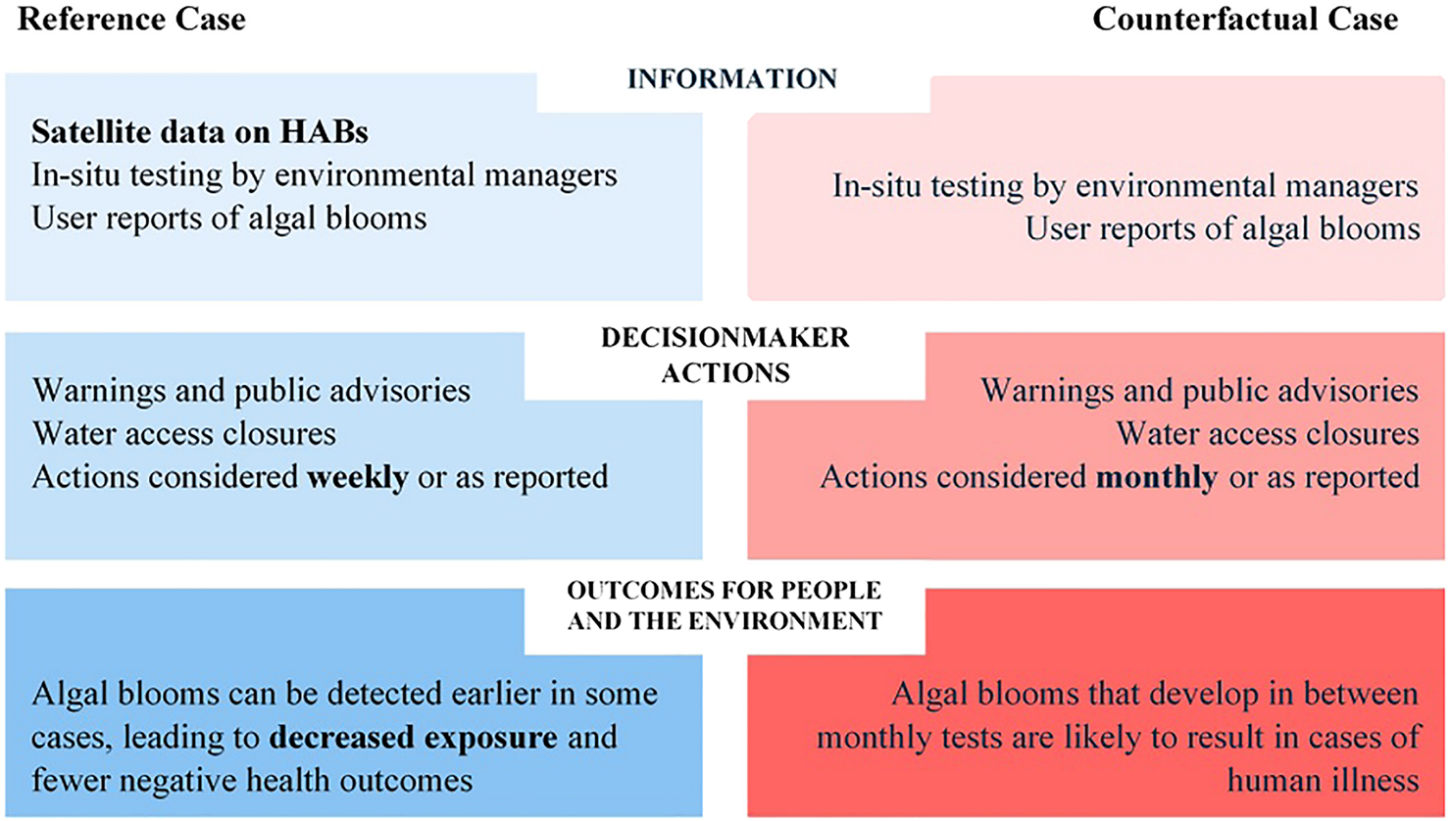
Impact assessment framework.

Figure 3 illustrates our definitions of the reference and counterfactual cases for analyzing the socioeconomic benefits of satellite information on cyanoHABs. The first column of the figure describes the information used by the decision maker, the decision maker's actions, and the socioeconomic outcomes of those actions in the reference case, that is, when satellite information is available. The second column describes the same elements for the counterfactual case, that is, when the only information available on cyanoHABs is from in situ testing and visitor reports.

The first row of Figure 3, labeled “Information,” illustrates the different information available to the decision makers in the reference and counterfactual cases. The decision makers in the reference case have access to satellite data on cyanoHABs, in addition to the in situ test information available in the counterfactual case. It is important to note that, in all real‐world applications that we are aware of, satellite data do not replace other forms of cyanoHAB detection; in the reference case, it is an addition to the in situ testing and visitor reports. Indeed, satellite data have a very specific contribution to the decision context. For example, the CyAN data can warn environmental managers of cyanoHABs that are developing in between monthly in situ tests or before visitors are aware of an event. As mentioned previously, CyAN cannot directly detect toxins or if the concentration of those toxins poses a human health risk (Stumpf et al., 2016). However, advisory actions can be considered more frequently with the introduction of a weekly monitoring system to supplement the traditional monthly in situ tests. Field and lab tests are still essential, at this time, to determine if a bloom is present and, more importantly, if it is producing toxins at concentrations that could pose a community health risk. In situ measures are also necessary for satellite algorithm validation and monitoring in smaller water bodies. The ability to consider advisory actions weekly with satellite data, in contrast to monthly with no satellite data, is reflected in the second row of Figure 3, labeled “Decision maker actions.”

The third and last row of Figure 3, labeled “Outcomes for people and the environment,” defines the difference in the key socioeconomic benefit that is linked to the decision context, which is human health. In the reference case, cyanoHABs might be detected earlier than in the counterfactual case, leading to decreased human exposure to toxins and fewer negative health outcomes. Overall, Figure 3 maps out the causal logic behind why the availability of satellite information on cyanoHABs can yield socioeconomic benefits in the form of enhanced protection of human health.

In the next section, we apply this framework to a specific cyanoHAB event in Utah Lake with the goal of obtaining a quantitative estimate of the magnitude of this improvement in human health outcomes. We note that other analytical approaches may also allow for the quantification the socioeconomic benefits of CyAN data tied to this event, such as a “replacement cost” approach that estimates the incremental cost of increasing field sampling from monthly to weekly frequency.

## 5 Application: Utah Lake CyanoHAB

### 5.1 Background

Forty miles south of Salt Lake City, Utah Lake is a popular destination for boaters, fishers, and nature enthusiasts. Warm western summers combined with the lake's shallow depth (14 feet at its deepest point) make Utah Lake highly susceptible to cyanoHABs. Algal blooms have occurred in Utah Lake in 2014, 2016, 2017, 2018, and 2019 (Utah DEQ, 2016). While blooms are not guaranteed to occur every year, environmental managers are constantly on the lookout for signs of cyanobacteria in the warmer months. Fortunately, at 148 square miles, the lake is large enough to take advantage of the CyAN satellite data from Sentinel‐3 OLCI.

In the summer of 2017, Utah's department of environmental quality conducted the usual monthly tests to monitor for cyanoHABs in Utah Lake and other water bodies across the state. On 12 June, in situ tests revealed no cyanobacteria present on the lake. However, on 20 June, satellite images from CyAN revealed a biomass developing in Provo Bay, on the eastern shore of the lake. Lab tests revealed that cyanobacteria were producing toxins in the bloom, and warnings were promptly posted around the affected area informing recreational users of the danger associated with exposure to toxic cyanoHABs. A press release was also issued, informing citizens of the bloom on Utah Lake. Close monitoring of the cyanoHAB continued until November, when the event was considered over, and regular monthly testing resumed.

The cyanoHAB in Utah Lake provides an excellent opportunity for applying the impact assessment framework described in section 4 because Utah has a well‐documented procedure for detecting and responding to cyanoHABs. In order to apply the framework to this specific cyanoHAB event, we conducted interviews with managers from the Utah Department of Environmental Quality to develop a more detailed characterization of the decision context in which information from CyAN were used. We supplemented these interview findings with existing results from the public health literature and statistics from park visitor and tourism records. This resulting framework informed the use of an empirical approach for the impact assessment that consists of the following four steps: (1) estimate the amount of time between the cyanoHAB developing and a recreational advisory being posted in the absence of CyAN; (2) estimate the number of people who would have made contact with the water after the bloom developed and prior to the advisory being posted; (3) estimate the number of people who would have experienced symptoms after contact; and (4) estimate the cost per case of human illness resulting from the cyanobacterial bloom. Together, these figures provide an estimate for the cost of illness avoided due to the availability of CyAN satellite data, which can be interpreted as a socioeconomic benefit of using the satellites to manage this cyanoHAB event.

### 5.2 Estimating Reductions in Exposure

The prompt posting of a recreational advisory in the reference case is the foundation for the argument that satellite data provide socioeconomic benefits. Therefore, obtaining a credible estimate of when the advisory would have been posted in the counterfactual case is critical, though obviously challenging given we are unable to observe that scenario. Though the next monthly in situ test for Utah Lake was scheduled 3 weeks after satellite detection, it is unlikely that the cyanoHAB would have gone undetected by visitors, particularly if it was making people sick. Our best estimate for the delay in public awareness of the cyanoHAB is thus equal to the delay in the presentation of symptoms, plus any additional time needed to post warnings and advisories around the lake. In order to arrive at an estimate of the duration of this delay, we refer to Pilotto et al. (1997), a study of 852 individuals who swam in a cyanobacterial bloom in Australia. This study finds that noticeable symptoms began to develop in these individuals 2 to 7 days after contact with a toxic cyanoHAB. We thus estimate that 7 days would serve as a reasonable exposure window; it is reasonable to assume that this length of time would be sufficient for individuals to develop symptoms, determine that they were caused by the cyanoHAB, contact the relevant authorities, and allow the authorities to post proper signage.

This estimate is supported by historical evidence. In 2016, just 1 year prior, a bloom developed in Provo Bay on Utah Lake. The Utah Poison Control Center fielded hundreds of calls from people reporting symptoms after swimming or boating in the lake. It took the Utah Department of Environmental Quality approximately 3 days to post advisories and warnings around the entire lake (Utah DEQ, 2016). The peak in calls was 1 day after the bloom was initially reported, 14 July. Calls reporting symptoms continued into the weeks following the bloom, indicating some late presentation of symptoms or delay in signage.

To determine how many people avoided exposure to the toxic cyanoHAB during that 7‐day period in the reference case, we referenced historical park visitor data for Utah Lake State Park and visitor surveys that recorded the types of recreational activities visitors engaged in. Historical data from the state park indicate that approximately 10,000 people visit the park per week during the month of June (Utah State Parks, 2020). In addition, a 2013 community survey by Utah County found that 21% of park visitors swim during a park visit, 51% do some kind of boating, and 22% do watersports like wakeboarding or waterskiing (ULWQS SC/SP Meeting, 2017). Because there can be overlap between these three groups and we are most interested in individuals who swim or participate in watersports, we estimate that about 40% of park visitors participate in activities that can expose them to an active cyanoHAB.

While these data provide a good estimate of how many individuals may have avoided exposure at Utah Lake State Park, Utah Lake has several other popular access points where visitors might have been exposed to the cyanoHAB. To supplement our data from Utah Lake State Park, we analyzed Google Reviews for each marked public access point for Utah Lake. Google collects reviews on a voluntary basis for all designated “locations” on its mapping service. We assume that the number of reviews for a specific location is a function of the number of visitors to that location. Using the data from the state park as a baseline, we estimate how many visitors might have used the other access points, assuming the proportion of reviews a location has is reflective of the proportion of visitors. As illustrated in Table 1, we find that Utah Lake State Park accounts for approximately half of all Google Reviews for lake access points. Therefore, we assume that the state park accounts for approximately half of all visitors to the lake, yielding the estimated visitors per week listed in Table 1. Overall, we estimate that about 9,000 people would have been exposed to the lake in 1 week, without cyanoHAB warnings or advisories. Our assumption that Utah Lake State Park accounts for half of all visitors is likely conservative, since the state park offers a wide range of recreational activities and some access points are more uniquely designed for water access.

**Table 1:** Estimation of Visitors Based on Online Reviews of Access Points

Access point	Reviews	Proportion of reviews	Estimated visitors (1 week)
Utah Lake State Park	730	.43	4,000
Shore Trail	91	.05	452
Vineyard Beach	68	.04	361
Public Access Point	39	.02	181
Hot Springs	141	.08	722
Eagle Park	66	.04	361
Lincoln Beach	129	.08	722
Sandy Beach	47	.03	271
American Fork Beach	84	.05	452
Lindon Marina	284	.17	1,535
Powell Slough Park	35	.02	181
Total	1,714	1	9,032

*Note*. Estimates based on an exposure rate of 4,000 people per week at Utah Lake State Park during the summer.

We then narrowed the scope of the estimate to access points near Provo Bay, the source of the cyanoHAB. Since Hot Springs and Eagle Park access points are on the western shore of Utah Lake, we eliminated those access points from our calculation, since people using those access points near the beginning of the bloom event would likely not have gotten sick. Therefore, our final estimate of visitors that would have been exposed to the cyanoHAB in the counterfactual case is 8,000.

### 5.3 Estimating Reduction in Human Health Impacts

We focus on two quantitative components of human health outcomes: the rate of symptom presentation and the distribution in severity of symptoms. We reviewed the public health literature and other sources to estimate the proportion of Utah Lake visitors who would have been exposed to the cyanoHAB and would have gotten sick without appropriate warnings from Utah DEQ. While this existing literature draws a definitive connection between toxins and human illness, there is little empirical evidence that would help us obtain quantitative information on the rates at which individuals exposed to cyanoHABs experience human health impacts.

The World Health Organization has officially documented three known events when human illness was attributed to cyanotoxins in recreational water (World Health Organization, 2003). In 1989, 10 out of 20 English soldiers became ill after swimming and canoe training in water with a significant cyanobacterial bloom. Two soldiers developed severe pneumonia and required hospitalization. Swimming skills and amount of water ingested appeared to be correlated with the severity of symptoms. In 2010, an Ohio resident was exposed to dense scum of cyanobacteria while washing off his dog, which had swum in the bloom. The dog died within a few days, and the man was hospitalized a few weeks later with ulcers in his eye, slurred speech, stomach problems, and inability to feel the left side of his body (Marshall, 2012).

Additionally, the Centers for Disease Control (CDC) studied disease outbreaks associated with cyanoHAB outbreaks in 2009 and 2010 in New York, Ohio, and Washington. The CDC determined that 11 outbreaks occurred across during those years. During the 11 outbreaks, 61 illnesses were reported, with two hospitalizations and zero deaths. The study recorded several different health effects due to exposure in recreational freshwater and concluded that the nonspecific nature of symptoms makes it particularly challenging for health care providers to identify and diagnose cyanoHAB‐associated illnesses (Hilborn et al., 2014).

To overcome this symptom identification challenge, we used the findings of Stewart, Webb, Schluter, Fleming, et al. (2006). In this study, researchers observed recreational users in Australia and Florida who were exposed to toxins to determine how often people experience symptoms and the distribution between mild and severe symptom presentation. This research was specifically focused on low cell surface area cyanobacteria exposure, which is typically associated with lower levels of symptom presentation. The authors find that about 5% of recreational users exposed to cyanoHABs experienced some gastrointestinal symptoms. About 3% experienced ear symptoms, 6% experienced eye symptoms, and 15% experienced some respiratory symptoms. About 50% of those individuals experienced gastrointestinal symptoms categorized their illness as moderate or severe. The 5% figure for gastrointestinal illness is the most reliable estimate that we were able to find for the rate at which individuals exposed to cyanoHABs experience human health impacts, because it uses a group of swimmers not exposed to cyanobacteria to control for the average presentation of gastrointestinal symptoms. Stewart, Webb, Schluter, Fleming, et al. (2006) also found that gastrointestinal symptom presentation was split evenly between mild symptoms and more moderate and severe symptoms. Using our earlier exposure estimate for the counterfactual case (8,000 people exposed), we project that approximately 200 people would have experienced mild symptoms and 200 people would have experienced moderate or severe symptoms.

Stewart, Webb, Schluter, Fleming, et al. (2006) does not distinguish between cases with moderate and severe symptoms. However, the difference in societal costs associated with these two levels of symptom severity may be substantial. To obtain an estimate of how many of the estimated 198 people who would have experienced moderate or severe symptoms would have experienced severe symptoms, we use information from cyanoHAB‐associated outbreaks documented by Hilborn et al. (2014). In 11 outbreaks that occurred between 2009 and 2010 in New York, Ohio, and Washington, the authors find that 61 people experienced symptoms, of which 3% required hospitalization and 12% went to some sort of emergency department to seek diagnosis and treatment. Based on this information, and taking a conservative approach, we estimate that of the 198 people who would have experienced moderate or severe symptoms, 30 would have experienced severe symptoms. We vary this number in our sensitivity analysis to account for the uncertainty related to the number of severe cases.

Evidence from the cyanoHAB event in 2016 further supports these figures. On 15 July, 225 people called into the poison control hotline to report the algal bloom prior to an advisory being posted (Utah Poison Control Center, 2016). Over the next couple of weeks nearly 300 additional callers reported the bloom (Utah Poison Control Center, 2016). For the case we investigate in 2017, when the HAB was detected via remote sensing, no reports were made to the Utah Poison Control Center in the initial weeks of the bloom. For the entire month of June, only 18 reports were made to the Utah Poison Control Center (Utah Poison Control Center, 2017). Furthermore, between 2016 and 2019, the Poison Control Center reports symptoms in 23% to 32% of callers, with gastrointestinal symptoms causing most complaints. While these symptom presentation rates are higher than the 5% figure derived from Stewart, Webb, Schluter, Fleming, et al. (2006), there are two main considerations that support the use of a lower rate in our calculations. First, the Poison Control Center rates are based on information collected from exposed individuals who called in, who are more likely to have experienced negative symptoms relative to the all individuals who were exposed. Second, the Poison Control Center rates include all symptoms, not just gastrointestinal illness.

Although respiratory symptoms comprise a substantial portion of symptom presentation in the above studies, we did not examine the societal benefits of avoiding respiratory symptoms because the cost estimates in the literature for treating this class of symptoms are often associated with illnesses such as lung disease or asthma, which are much more costly to treat than the illness associated with cyanoHAB exposure. Hoagland et al. (2009) estimates that *Karenia brevis* blooms in Florida cost society approximately $513 dollars per case of respiratory illness. However, this calculation assumes 3 days of lost productivity and a doctor's visit in most cases, which is likely more severe than many cyanoHAB exposure cases. We determined that the literature on gastrointestinal illness was more applicable for our research, but it is important to note that excluding the costs of these other symptoms means our estimate of the socioeconomic benefit of satellite data represents a lower bound for the true magnitude of benefits.

### 5.4 Estimating Reductions in Cost of Illness

After settling on an estimate for the number of cases of gastrointestinal illness that would have occurred in the counterfactual case, we reviewed the health economics literature to find an estimate for the cost to society for this type of illness. When someone experiences illness, they pay for medication, doctor's visits, and missed work. Depending on the severity of the illness, hospitalization costs might also need to be quantified. These costs are borne by society in the counterfactual case. However, when satellite data were used to detect the cyanoHABs early on, these costs were avoided, and thus represent socioeconomic benefits.

The literature on the cost of illness related to cyanoHABs is limited, so we relied on the more expansive health literature that documents the typical costs associated with gastrointestinal illness. Because costs vary so dramatically with the severity of symptoms, we focused on research that separated cost estimates into different categories of illness. In one study focusing on illnesses contracted from recreational water use, DeFlorio‐Barker et al. (2018) find that the social costs of gastrointestinal illness range from $9.50 to $302,685.57 (in 2007 U.S. dollars). For a mild case, these costs are the amount needed to cover basic over‐the‐counter remedies and are estimated to be $9.50 per case. Moderate cases are those that include a doctor's visit and missed work, and costs are estimated to be $223.38 per case. Severe cases include hospitalizations and deaths. Because cyanoHABs have not been sufficiently linked to human fatalities, we chose to adjust the calculation for the cost of a severe case to exclude potential loss of life. After this adjustment, we estimate that the social cost of a severe case of gastrointestinal illness is approximately $9,067. Adjusted for inflation, mild, moderate, and severe cases cost $11.23, $264.38, and $10,719.02, respectively, in 2017 U.S. dollars. We weight these estimates based on evidence in the literature about the proportion of individuals that present mild, moderate, or severe symptoms, described in the previous section.

Other literature supports these approximate valuations. Henson et al. (2008) find similar figures for the cost of acute gastrointestinal illness in British Columbia, Canada. The study finds that severe cases cost approximately CAN$996.07 each ($905.80 in 2017 U.S. dollars), moderate cases cost approximately CAN$231.96 each (USD$210.94), and mild cases cost approximately CAN$122.23 each (USD$111.15). These estimates are more narrowly distributed than the DeFlorio‐Barker et al. (2018) estimates, but the resulting weighted estimate for the cyanoHAB exposure is on the same order of magnitude as our original estimates. While both studies analyzed medical costs and lost productivity to produce their estimates, we were inclined to use the DeFlorio‐Barker et al. (2018) estimates in our calculation since they more directly reflect our population of interest: Americans exposed to contaminated recreational water.

### 5.5 Estimate of Socioeconomic Benefits

**Figure 4:**
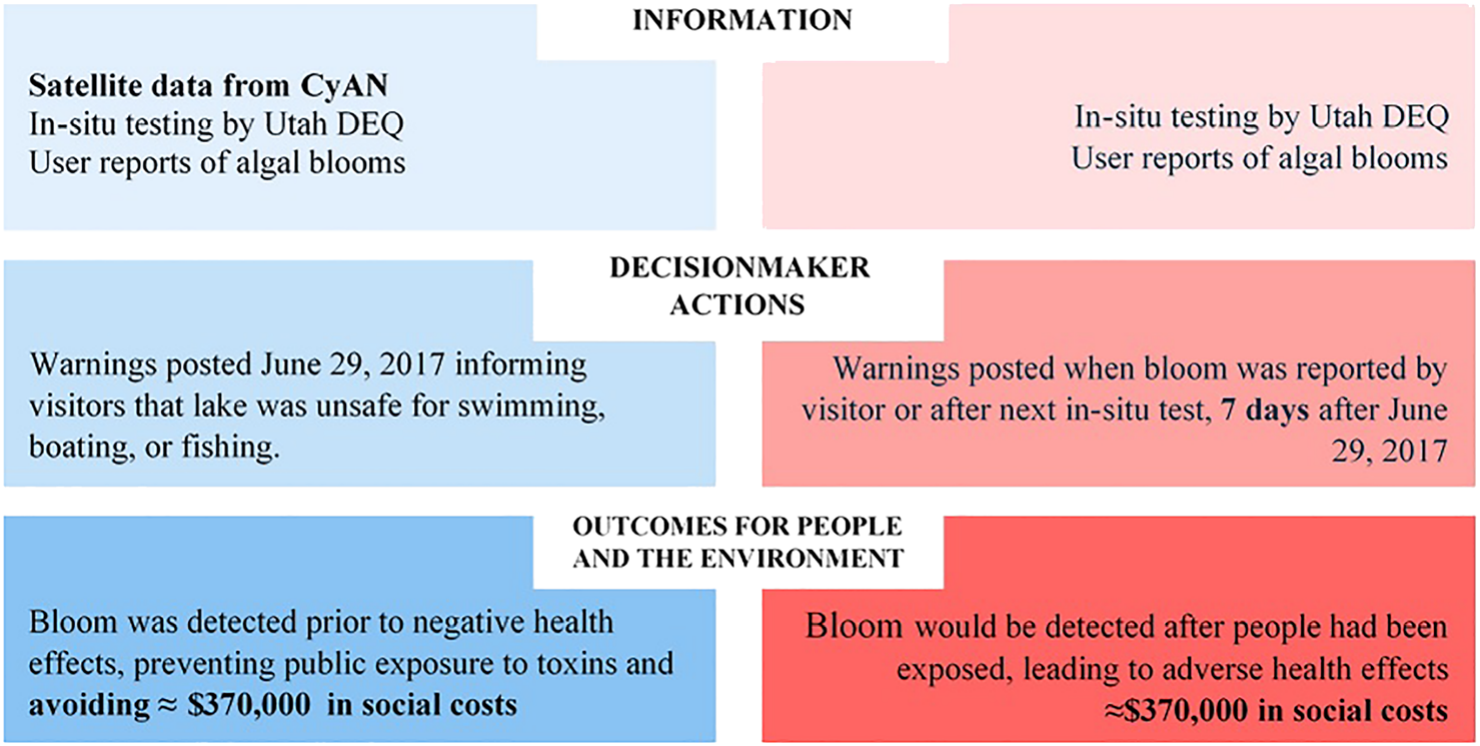
Impact assessment framework for 2017 Utah Lake cyanoHAB event.

In order to estimate the total reduction in cost of illness that can be attributed to the availability of satellite data in the 2017 Utah Lake event, we multiply the number of individuals who would have experienced symptoms at different levels of severity, derived in section 5.3, with the cost of treating the symptoms experienced at each level of severity, derived in section 5.4. Table 2 summarizes these results for the range of estimates we consider for exposure, presentation of symptoms, and cost of illness. We find that in the counterfactual case, approximately $370,000 in health care costs were incurred as a result of cyanoHAB exposure. Because there were no cases of illness in the reference case, we conclude that the satellite data provided approximately $370,000 in socioeconomic benefits to society. We summarize how this central estimate of the value of CyAN information in this cyanoHAB event was derived in Figure 4, which is a more detailed version of the impact assessment framework illustrated in Figure 3 that has been tailored for this specific application.

**Table 2:** Cost of Illness and Its Determinants in the Reference and Counterfactual Cases

Key figure	Reference case (satellite data)	Counterfactual case (no satellite data)
Time until advisory posting	0 days	7 days
Number of people exposed to cyanoHAB	0 people	8,000 people
Number of people who experience Symptoms	0 experience symptoms	200 experience mild symptoms 170 experience moderate symptoms 30 experience severe symptoms
Cost per case (2017 USD)	Mild: $11.23 Moderate: $264.38 Severe: $10,719.02	Mild: $11.23 Moderate: $264.38 Severe: $10,719.02
Total estimated Cost (2017 USD)	$0 in costs due to health outcomes	Approximately $370,000 in costs due to health outcomes

This estimate is sensitive to the estimates developed in sections 5.2 through 5.4 that define the time delay in posting an advisory, the number of people exposed to the cyanoHAB, the number of people who experience symptoms, and the cost per case of illness. Table 3 demonstrates the sensitivity of our socioeconomic benefit estimate to changes in our key figures based on alternate data sources or assumptions. Our estimate is particularly sensitive to changes in the timeline for advisory postings. If the cyanoHAB was detected by a visitor quickly, then societal benefits are substantially lower. In the unlikely event that regular use of the lake continued until the next in situ test (3 weeks after satellite detection), the societal benefits would nearly triple. The estimate is also very sensitive to the number of severe cases of gastroenteritis that result from the cyanoHAB. At about $10,000 in health care costs per case, small changes in the number of severe cases can create dramatic changes in the estimate of social benefits provided by early satellite detection. Our sensitivity analysis reveals a range in socioeconomic benefits of approximately $55,000 to $1,057,000 depending on our core assumptions and data choices.

**Table 3:** Sensitivity of Socioeconomic Benefits Estimate

Key figure adjusted	Adjustment	New figure	New estimate of social benefit^a^
Time until advisory posted	Reduced to minimum	2 days	$106,000
Time until advisory posted	Increased to maximum	20 days	$1,057,000
Number of people exposed to cyanoHAB	State Park only	4,000 people	$185,000
Number of people exposed to cyanoHAB	All access points included	9,000 people	$416,000
Cost per case (2017 dollars)	Henson et al. (2008) estimates used and adjusted for exchange and inflation	Mild: $111.15 Mod: $210.94 Sev: $905.80	$85,000
Number of people who experience symptoms	No severe cases	200 mild 200 moderate	$55,000
Number of people who experience symptoms	Double the severe cases	200 mild 140 moderate 60 severe	$682,000
Range of Alternative Estimates			$55,000–$1,057,000

a Rounded to the nearest $1,000 to represent the limited precision of some input values.

This sensitivity analysis illustrates variability in social benefit based on alternative estimates. However, our analysis is also limited by unobserved or unquantifiable variables that impact the socioeconomic benefits. First, we did not consider the full list of possible symptoms associated with cyanoHAB exposure, because there is such limited literature associated with the presentation of those symptoms after cyanoHAB exposure, their relative severity, and their socioeconomic costs. Second, we did not consider social damages to pets and livestock as a result of the cyanoHAB. As previously mentioned, cyanoHAB exposure can be fatal for animals. These lives certainly have value, and some studies have attempted to monetize these values (e.g., Carlson et al., 2019), but without a better understanding of how many pets would have been exposed, we decided it was best to leave these costs out of our analysis. Third, we assumed full compliance with the posted recreational advisories, which is unlikely. Advisory compliance rates vary with time, place, and perceived risk, so they are best applied by policy makers that understand the relevant population. The socioeconomic benefits scale linearly with the compliance rate, so integrating a compliance estimate would be straightforward using this impact assessment framework.

Finally, we did not calculate the costs to the local recreational economy associated with recreational advisories that limit lake access. Businesses that rely on tourism and lake visitors for customers would likely suffer financial setbacks as a result of decreased lake visitation. However, without understanding the relationship between decreased swimming and boating and the performance of these businesses, there was insufficient data to include in our analysis. Taken together, we assume that these factors would increase the overall estimate of socioeconomic benefit associated with using satellite data to supplement the cyanoHAB detection process.

## 6 Conclusion

Our impact assessment framework to estimate the socioeconomic benefits of satellite remote sensing for detecting cyanoHABs and managing recreational advisories at freshwater lakes, paired with an application to a 2017 cyanoHAB event in Utah Lake, provides evidence that satellite data can yield substantial socioeconomic benefits for communities that leverage remote sensing tools to supplement their cyanoHAB monitoring strategies. We document several existing decision contexts in which satellite data on cyanoHABs were used to inform specific management decisions. In addition, we present a general impact assessment framework that identifies differences between the actions that decision makers take when the satellite information is available and the actions they take when satellite information is not available. Applying this framework to the Utah Lake event, we find that using the CyAN satellite data to detect the cyanoHAB on Utah Lake likely saved the community hundreds of thousands of dollars in health costs by enabling early detection and the prompt posting of recreational advisories.

We acknowledge several limitations of the approach we take in the Utah Lake application, particularly as it applies to other cyanoHAB detection cases. Utah Lake is a massive recreational destination with thousands of annual visitors. The benefits of satellite data found in this study are not representative of most lakes in the country and are likely higher than the typical lake experiencing a toxic cyanoHAB due to the number of affected persons. Additionally, the estimate we provide is grounded in a specific temporal context. Public awareness of cyanoHABs historically has been low but is steadily increasing as toxic cyanoHABs become a more regular and pronounced threat. Indeed, as of 2019, environmental managers in Utah have decided to permanently post cyanoHAB warning advisories around Utah Lake, warning visitors not to swim or participate in water sports if they see signs of cyanobacteria. This decreases the potential value of future satellite detection of cyanoHABs, as it critically alters the decision context we illustrate in Figure 2.

This analysis is limited to evaluating the socioeconomic benefits delivered by cyanoHAB detection via remote sensing. We did not perform a detailed analysis of program costs due to challenges with attributing a portion of the costs associated with launching satellite missions or manufacturing sensors, which are used for many other purposes, to the particular scientific task of monitoring cyanoHABs. However, we can obtain a rough estimate of program costs, assuming that the costs of launching and operating satellites and sensors are sunk, by considering remaining costs which are primarily associated with data storage and hosting, maintaining and updating the data repository (including personnel costs), and making the data publicly available via a cloud‐based service. Assuming that about 200 Gigabytes of data need to be stored and processed per year (Schaeffer et al., 2015) at a rate of $0.40 per Gigabyte and maintaining and updating the data repository requires two GS‐12 equivalent scientists (at a rate of $70,000 per year), the cost for supplying a year's worth of satellite data is on the order of $141,000.

Finally, we did not consider any negative impact on recreational industries in the Utah Lake area. Research shows that cyanoHABs and cyanoHAB advisories can create millions of dollars in lost revenue annually for states that rely on tourism and recreation for business revenue (Hoagland et al., 2002). In a full benefit‐cost analysis of the CyAN satellite data or other remote sensing tools, it is important to consider these costs in addition to the benefits associated with reduced health risks provided by early cyanoHAB detection. This analysis would require significant additional collection of data on local recreational businesses, including information on revenues and costs of operation, a task we leave for future research.

Despite these limitations, the research we present has significant implications for the value of using satellites to detect cyanoHABs. Significant variation in water use, bloom frequency, bloom spatial extent, bloom magnitude, toxin concentration, access to satellite data, and the degree to which satellite data inform management decisions prevents us from projecting our results to a national scale. To try to place the societal benefits we measure from the Utah Lake event into context, we note a 2019 study by the Environmental Working Group (EWG) that finds that news reports of algal blooms are on the rise, and there were over 500 bloom events reported on in the United States in 2019 (Environmental Working Group, 2019). These blooms did not necessarily involve cyanotoxins and might not have impacted recreational users. However, the number of blooms reported annually indicates that remote sensing tools could deliver great value on a national scale.

## Conflict of Interest

The authors declare no conflicts of interest relevant to this study.

## Data Availability

All data underlying this paper are available at the Mendeley Data (http://data.mendeley.com) repository at the following persistent identifier: https://doi.org/10.17632/vfwnvk2nr3.1. The files associated with this data set are licensed under a Creative Commons Attribution 4.0 International license. Registration or fees are not required to access the data.
